# Complexity of the International Agro-Food Trade Network and Its Impact on Food Safety

**DOI:** 10.1371/journal.pone.0037810

**Published:** 2012-05-31

**Authors:** Mária Ercsey-Ravasz, Zoltán Toroczkai, Zoltán Lakner, József Baranyi

**Affiliations:** 1 Interdisciplinary Center for Network Science and Applications (iCeNSA) and Department Physics, University of Notre Dame, Notre Dame, Indiana, United States of America; 2 Faculty of Physics, Babes-Bolyai University, RO-400084 Cluj-Napoca, Romania; 3 Department of Food Sciences, Budapest Corvinus University, Budapest, Hungary; 4 Institute of Food Research, Norwich Research Park, Norwich, United Kingdom; INSERM & Universite Pierre et Marie Curie, France

## Abstract

With the world’s population now in excess of 7 billion, it is vital to ensure the chemical and microbiological safety of our food, while maintaining the sustainability of its production, distribution and trade. Using UN databases, here we show that the international agro-food trade network (IFTN), with nodes and edges representing countries and import-export fluxes, respectively, has evolved into a highly heterogeneous, complex supply-chain network. Seven countries form the core of the IFTN, with high values of betweenness centrality and each trading with over 77% of all the countries in the world. Graph theoretical analysis and a dynamic food flux model show that the IFTN provides a vehicle suitable for the fast distribution of potential contaminants but unsuitable for tracing their origin. In particular, we show that high values of node betweenness and vulnerability correlate well with recorded large food poisoning outbreaks.

## Introduction

By 2030, food demand is expected to increase by 50% [1] and thus the global food supply is playing an increasingly critical role in the economical and political landscape [2], [3]. The latest deadly food poisoning outbreaks in 2011 (*Escherichia coli* in Germany [4], *Listeria monocytogenes* in the US [5]) and their economic, political and social effects clearly illustrated the importance of prompt tracing of the origin of specific food ingredients. This task is placing a huge pressure on regulation and surveillance.

**Figure 1:**
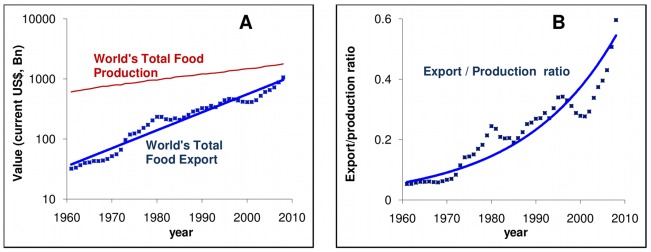
The world’s food trade grows faster than the food production. (**A**) (Log-linear scale). The world’s food production (thin red line), measured in current Billion US$, doubles in ca. 30 years, while the amount of food transported on the IFTN (linearly fitted small squares, blue) increases by ca. 10-fold in the same time. (**B**) (Linear scale). Food ingredients flow at an increasing rate from countries to countries, as shown by the exponentially increasing [world export]/[world production] ratio calculated from the above data (small squares fitted by an exponential curve). Note that this ratio is unaffected by the US$ inflation rate. Data obtained from UN databases [6], [23].

Since the 1960-s, global food transport has been increasing at an exponential rate, faster than food production itself, as illustrated in Fig. 1, which was generated using ComTrade [6], an agro-food import-export database of the United Nations (UN). The picture becomes even more complex if we factor in the growing number of countries relying on international food trade and, additionally, the fact that the traded food types have been increasingly moving from agricultural raw materials and staples towards processed and branded products. As a consequence, food fluxes between countries form a complex, dynamic web of interactions referred here as the International Agro-Food Trade Network (IFTN). For several countries, this web ensures access to any food item regardless of season and location. However, it may also present serious vulnerabilities [7], [8], [9]. As we show here, the IFTN has become a densely interwoven complex network [10], [11], [12], [13], creating a perfect platform to spread potential contaminants with practically untraceable origins.

**Figure 2:**
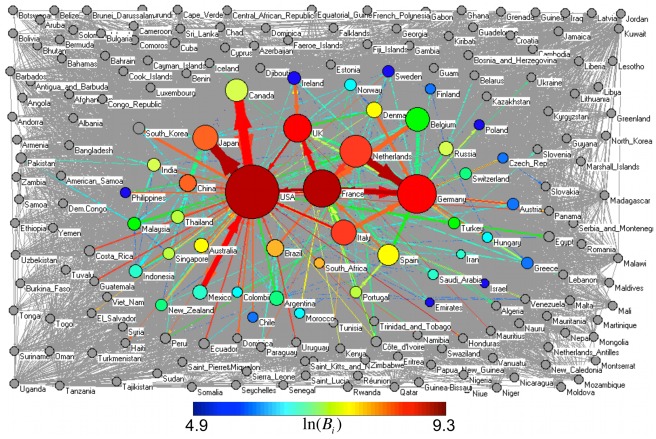
The complete International Agro-Food Trade Network in 1998. The IFTN is based on reported export, involving N = 207 countries (nodes) drawn as disks and M = 10645 trade fluxes (those worth more than 1 million US$), drawn as directed edges/links. The top 44 countries with the largest total trade activity (import+export) and the top 300 largest food-trade fluxes were colored according to their betweenness values (see Materials and Methods). The rest of the countries and edges are drawn with gray. The sizes of the colored disks are proportional to the logarithm of their total trade activity, ln(*E_i_+I_i_*). The thickness of the directed links is proportional to the log value of the trade flux in that direction, ln(*Φ_ij_*). The structure of the IFTN was similar throughout 1998–2008.

Using the ComTrade database [6], we constructed the IFTN and analyzed its structure and dynamics during the last ten years. Fig. 2, based on the 1998 data, shows a typical picture of the IFTN. The nodes of the network represent the countries, while the directed and weighted edges indicate the food trade fluxes between the countries. The magnitude of a flux (edge weight) represents the total value of the annual agro-food trade expressed in current US dollars (US$) from one country to the other. The size of a node is drawn proportional to the total import-export value of the country, while the thickness of an edge is proportional to the log-value of the food-flux it represents. Colors indicate the betweenness-centrality values of the nodes and edges as detailed in the caption of the figure and in the Materials and Methods section.

## Results

### General Trends and Structure of the IFTN

**Figure 3:**
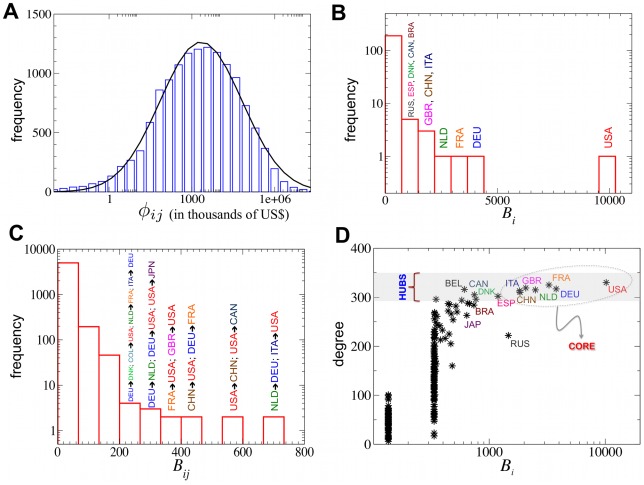
Structural properties of the IFTN. (**A**) Histogram of fluxes (blue bars) fitted by a lognormal distribution (solid continuous line). The parameters of the fitted distribution for ln*Φ* are *μ* = 7.68 (mean) and *σ* = 3.42 (standard deviation). The flux *Φ* is expressed in thousand US$ units. (**B**) Histogram of the betweenness centrality values of nodes and (**C**) edges. (**D**) A scatter-plot of degree *vs.* betweenness for every country. The figures represent the 2007 dataset.

The total amount of food-flux in the IFTN grew from 438Billion (B) US$ in 1998 to 1060B US$ in 2008; a 2.3-fold increase, while the total food production grew only 1.4-fold in the same period (from 1,400B US$ to 1,780B US$). The density of the IFTN increased from 25% in 1998 to 33% in 2008 (see the Materials and Methods). Unlike homogeneous random graphs, the IFTN has a broad degree distribution, indicating a heterogeneous network structure [10], [11]. The distribution of fluxes (number of edges with flux values within a given range) can be approximated by a lognormal distribution (Fig. 3A), implying that this distribution is also broad, with a fat tail.

A frequently used measure in the structural analysis of complex networks is the node- or edge-betweenness centrality (see [11], [14], [15] and also Materials and Methods). It quantifies how “central” is the position of the node/edge in the network, in the sense that high centrality nodes/edges collect large portions of the traffic through the network. For this reason, they also present the Achilles’ heel of a network as changes in the status of these nodes and edges will have the largest effect on the whole system [16], [17], both in connectivity and transport properties. Nodes with top centrality values play a critical role in the IFTN because any food-born substance (e.g. chemical or microbiological contamination) will spread most efficiently through them into the rest of the network, while tracing the source of such a substance is difficult due to the large number of network paths running through these nodes. Fast spread is also facilitated by the small value of the average shortest path (measured in hop-counts) of the IFTN, which is *L* = 1.52. That is, on average, one can reach any node in less than 2 hops from any other node along shortest trade routes. Though a single, specific food ingredient may not necessarily follow the shortest paths in the IFTN (e.g., it could be included into more complex foods and sent on various routes), the small value of the average shortest path length is still an indicator of the close proximity of almost all the nodes, guaranteeing fast spread on the network.

Figs. 3B,C present histograms of betweenness values for nodes and edges, respectively. These distributions show that the network is dominated by a centrally positioned small set of countries (shown with their 3-letter codes on the figures) and their trade relationships. Interestingly, despite its relatively small size (compared to other high betweenness countries such as USA or Germany), The Netherlands, with trades totaling 50B US$ in imports and 79B US$ in exports in 2008, has assumed a top centrality position over the years.

Fig. 3D plots all countries by their degrees and the corresponding betweenness values. It shows that countries with high betweenness also tend to be *network hubs* in the IFTN, *i.e.* they tend to have the largest degrees. However, there are also high degree countries that do not have high betweenness centrality values (*e.g.* Belgium). Note the role of Russia as a “bridge”-node, with a relatively high centrality, but a lower degree. Fig. 3D also reveals a core group of 7 nodes (within the oval in the picture), each engaging in trade relations with at least 77% of all the world’s countries. When combined, they are responsible for 30% of the total trade flux. These 7 nodes present hotspots for the whole of the IFTN, as changes in their status would generate the largest global impacts.

**Figure 4:**
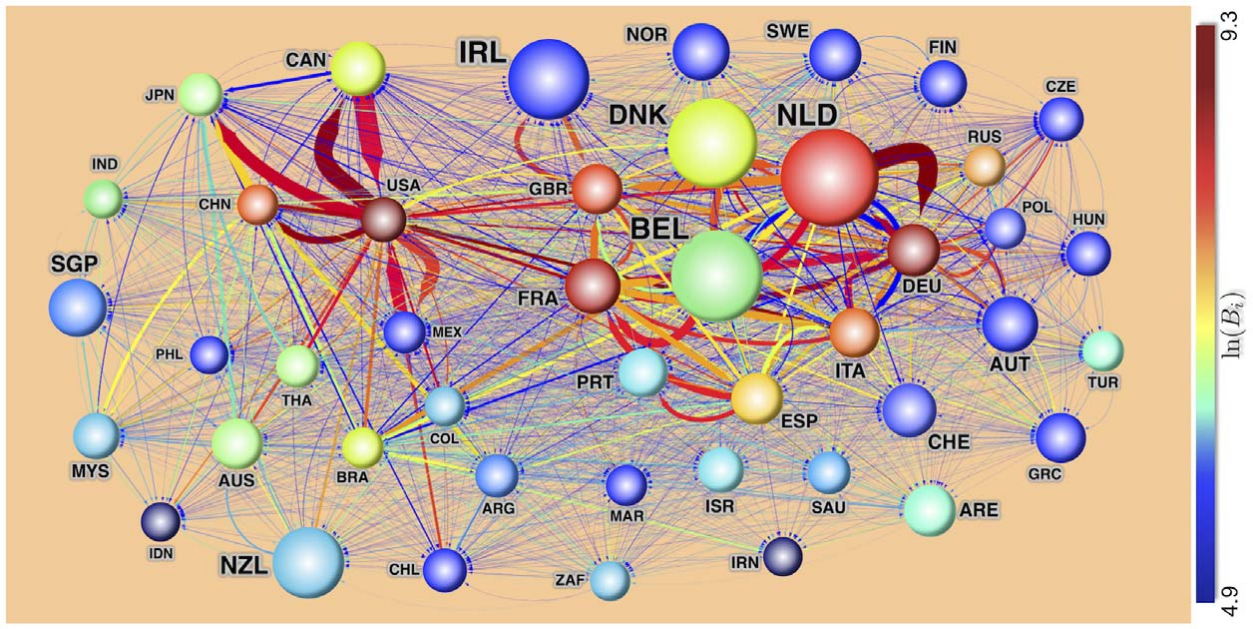
The backbone of the IFTN based on the 2007 dataset. The backbone is formed by the top 44 nodes (countries) with the largest total trade activity (import+export). Nodes and edges are both colored by their betweenness values; the thickness of the directed edges is proportional to the natural logarithm of the trade value in that direction, as in Fig. 2. The size of a node, in this figure, is proportional to the logarithm of the *per capita* trade activity, *i.e.* ln[(*E_i_*+*I_i_*)/*Π_i_*] where *Π_i_* is the population size of the country *i*. Countries are labeled by their 3-letter ISO 3166 codes.

Fig. 4 shows the backbone of the IFTN in 2007. The nodes are colored according to their betweenness centrality values, just as in Fig. 2, but here the node size is proportional to the logarithm of the total import+export value *per capita* in that country. Although USA has the largest betweenness value, the per-capita trade activity is largest for The Netherlands. Therefore, combining this with the fact that it has the 4-th largest betweenness, the food traders of The Netherlands have probably made their country into the most critical hub of the IFTN. Assuming that this overall picture of the network is sufficiently representative for food products that may act as suitable vectors for microbiological or chemical contaminations, the products that start from or go through The Netherlands would most efficiently affect the whole system.

### Spread and Tracing on the IFTN

The above observations made about vulnerabilities are based on graph-theoretical properties of the IFTN. Next, we develop a dynamic model, by tracking the food fluxes between the countries (Food Flux Model, or FFM), which will further underscore the potential of the IFTN to efficiently spread contaminants, and the poor outlook for their traceability. For brevity, in what follows, by “contaminated food” we mean a food item that contains some specific substance (such as chemical or microbiological contamination but it could also be common additives, or a subset of ingredients) to be followed or traced along the food trade pathways.

**Figure 5:**
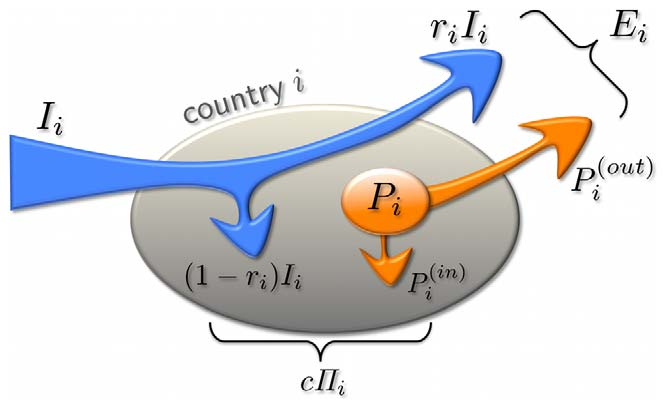
Schematics for the Food Flux Model. Country *i* with population of *P_i_* has a total yearly agro-food import *I_i_* , out of which *r_i_I_i_* is exported, and (1*-r_i_*)*I_i_* is consumed locally. A specific food ingredient to be tracked is produced in this country in the value of *P_i_* from which *P_i_^(out)^* will be included into its total export *E_i_* , while *P_i_^(in)^* is consumed locally. The parameter *c* represents the average value (in US$) of the food consumed by a person in a year.

The total import (export) into (from) a country *i* can be written as: 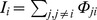 and 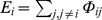 respectively. Suppose that a country *i* produces an amount of *P_i_* of a certain food, out of which *P_i_* ^(*in*)^ is consumed there, while the rest *P_i_* ^(*out*)^ is exported (Fig. 5), and thus *P_i_* = *P_i_* ^(*in*)^ +*P_i_* ^(*out*)^. Let *r_i_* denote the fraction of the imported food, which is passed on to other countries (via resale, repackaging, or after processing it into more complex food items). We can estimate the *r_i_* fractions as follows. The fraction of imported and produced food that is locally consumed (obtainable from the FAOSTAT food balance sheets [18]) can be written as *α_i_* = (1–*r_i_*)*I_i/_P_i_* ^(*in*)^. If we assume that consumption is proportional to the size of the population of a country (at least for the highest trade activity countries shown in Fig. 4), we can write: (1–*r_i_*)*I_i_*+*P_i_* ^(*in*)^ = *cΠ*_i_, where *c* is the typical value (in US$) of food consumed by a person in a year in the country *i* with a population of *Π_i_*.

We estimated the value of *c*, for the backbone of the IFTN, to be at around 10^4^ US$. This value is fairly constant over the backbone-countries. The reason is that these countries are approximately on the same level of economic development, and there is a low degree of variance between the shares of foods. (The analysis can, of course, readily be repeated with country-specific *c* values). From the above two equations it follows that.
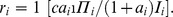



Suppose that a country *s* produces an amount of *D_s_* from a specific food ingredient and passes it to its neighbors in the IFTN at rates proportional to the food-fluxes (*Φ_sj_*) towards those neighbors. A fraction *r_j_* of that “contaminated” food, namely *r_j_D_s_Φ_sj_*/*E_s_* is then exported towards the neighbor *j*, while the rest, (1–*r_j_*)*D_s_Φ_sj_*/*E_s_* is consumed locally. The following recursion then describes the way food ingredients spread on the IFTN:
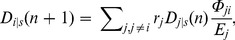
(1)where *n* denotes the number of export steps, *D_i|s_*(*n*) the amount (in dollar value) of food containing the ingredient in question, arriving into country *i* on the *n*-th step (this also allows for re-appearance in the same country), given it started from country *s*, and the summation is over all the neighbors *j* of *i* in the IFTN. The amount of food 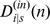 consumed in country *i* that contain the tracked ingredient can be obtained from the recursion



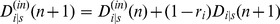
(2)The initial conditions for (2) are given as *D_i|s_*(0) = 0 if *i≠s* and *D_s|s_*(0) = *βE_s_*, where *β* represents the contaminated fraction of the export from country *s.* We chose 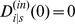 for all *i*.

We simulated and recorded the contamination spread for *n* = 5 steps, from every one of the top 44 countries with the largest trade activity as shown in Fig. 4. After the simulations, we selected the top ten (*s,t*) source-target pairs with the largest contamination *D_t|s_*(*n*) at the target country; see Table 1. Germany came out with the largest potential for contaminated food import with The Netherlands as the source of the contamination.

**Table 1:** Largest *D_t|s_*(*n*) contamination values and the respective source-target pairs using the 2007 dataset in the Food Flux Model.

SOURCE	TARGET	*D_t|s_*(*n*) (Million US$)
**The Netherlands**	**Germany**	**6.48**
**USA**	**Japan**	**6.46**
**Canada**	**USA**	**6.05**
USA	Canada	5.24
USA	USA	4.97
France	Germany	4.93
The Netherlands	UK	4.63
Germany	Germany	4.24
The Netherlands	France	4.40
The Netherlands	USA	4.02

Parameters used for the simulation: *β* = 0.001 and *n* = 3.

**Figure 6:**
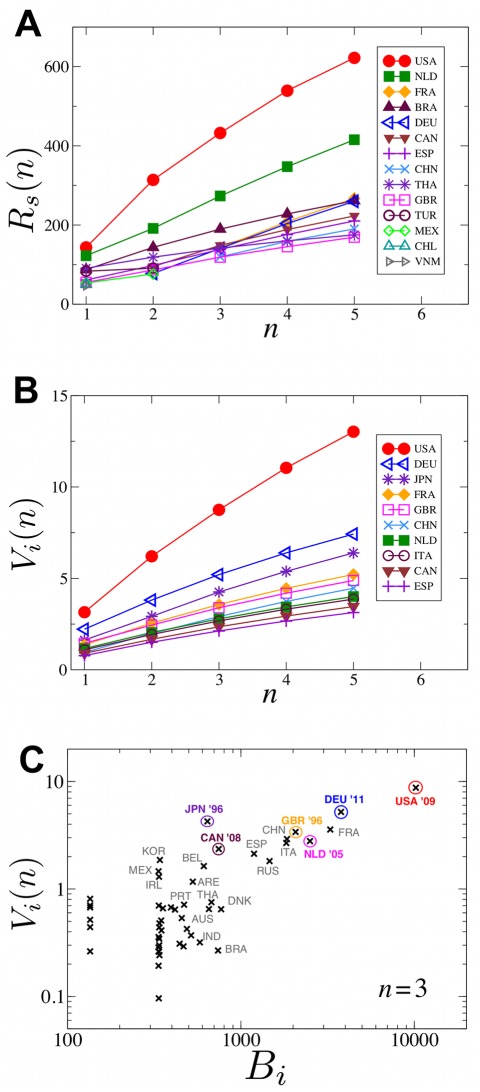
Spread analysis based on the Food Flux Model. **(A)** Evolution of the contamination impact *R_s_*(*n*) and **(B)** vulnerability *V_i_*(*n*), for the top 14 countries as function of the export steps, *n*. **(C)** “Vulnerability *vs.* betweenness” scatter plot for the 44 countries with the largest trade activity. Countries with significant food poisoning cases in the last 15 years are indicated by encircled symbols. In particular: the 2011 *Listeria* outbreak in the USA, from produce, causing 29 deaths [5]; the 2011 *E. coli* outbreak in Germany, from red beet sprout, with 46 deaths and 4000 diagnosed cases [4]; the *Salmonella* outbreak in 2005 in The Netherlands with 165 diagnosed cases [24]; the 1996 *E. coli* outbreak in the UK with 512 confirmed cases, 17 deaths [25]; the 2008 *Listeria* outbreak in Canada with 57 diagnosed cases and 27 deaths [26]; the 1996 *E. coli* outbreak in Japan, from radish sprout, with 2 infant deaths and more than 5000 hospitalized [27].

Considering the above mechanism as a *worst-case scenario*, we may assess how a contamination starting from a source country *s* affects the *global population*. This can be quantified via 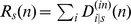, which we call the contamination impact. Fig. 6A shows the top 14 countries by their *R_s_*(*n)* value, as function of export steps, *n*.

We can also define a vulnerability measure *V_i_(n)* for a country *i*, as the *average impact* generated by other countries as if the contamination started from there, where the average is taken over all sources *s*. That is 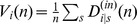 (Fig. 6B). As seen in Figs. 6A,B, the ranking for the countries with the highest impact and vulnerability values is practically independent of *n,* the number of export steps. As the diameter of the IFTN is small, contamination can spread very efficiently, and thus already modest values of *n* will start capturing the effects on the whole network.

The betweenness-based top lists in Figs. 3C,D correlate well with the top lists of Figs. 6B,C, which was obtained using the FFM. In particular, the USA, The Netherlands and Germany repeatedly emerge among the top hotspots for contamination impacts. Fig. 6C shows a scatter-plot of “vulnerability *vs.* betweenness” for the 44 countries studied. Encircled symbols show that high vulnerability and betweenness values (see Table 1) correlate well with recorded large food poisoning outbreaks.

Note that, although our predictions are based on coarse data, the developed models can be certainly refined once higher resolution data (food types, time-scales etc) becomes available.

## Discussion

The World Trade Web (WTW) has been extensively analyzed by network methods, for example in [19] and [20]. Our aim was not to repeat it for a subset of the WTW, but to demonstrate that the trends shown in Fig. 1 cannot be sustained if both free trade and the demand for biotracing are to be met. During a food poisoning outbreak the first and most important task is to identify the origin of the contamination. Delays in this task can have severe consequences for the health of the population and incur social, political and economical damages with international repercussions. A case in point is the consequences of the three weeks delay in identifying the origin of the *E. coli* contamination in Germany in June 2011 [4].

Note that our study *does not predict* an increase in the number of food poisoning cases but that, when it happens, there will be inevitable delays in identifying the sources due to the increasingly interwoven nature of the IFTN. That is, even if food contamination was less frequent, for example due to better local control of production, its dispersion/spread is becoming more efficient. In particular, our study identifies critical spots in the network that may seriously hamper future biotracing efforts. Although the analysis presented here is based on coarse data representing aggregated food fluxes, it can also aid with biotracing, in a “Bayesian approach” sense by providing a list of *most probable* sources and pathways to be used as starting points.

Recently there have been calls for an interdisciplinary approach [*7*] to monitor, understand, and control food trade flows as it becomes an issue no longer affecting just single countries, but the global livelihood of the human population. Such an approach would facilitate a better understanding of the IFTN, especially if it is broken down into time-scales, food types and their interdependencies. This would: 1) contribute to protection against outbreaks and intentional attacks; 2) help devise better traceability methods and thus increase consumer confidence; 3) allow for a better distribution of food and thus reduction of wastage [21], estimated to be about 30 – 40% globally [2]; 4) increase the reliability and stability of supply systems; and 5) help decrease the environmental burden of food production and distribution logistics. Such an interdisciplinary approach is entirely within the means of the state-of-the art of science and technology, if supported by detailed and systematic data collection. The role of state and interstate organizations (e.g. EU, UN) is essential in this. Although much of the food commerce and trade happens through the private sector, information collection and sharing should be incentivized to generate the data needed for an in-depth knowledge of the structure and dynamics of the IFTN, to ensure the safety and security of the global food system.

## Materials and Methods

### Data Sources and Analysis

The data used for this study was obtained from the ComTrade web site of the UN [6]. The HS-02 classification system was used to select the product codes 01–24, in the query, as these are related to food. The records used in our study were those reporting the values of certain food categories imported/exported from one country to another, expressed in current US dollars (US$). Since the total (worldwide) import must equal the total export, these were also compared/checked and no significant differences were found. Our calculations are based on records when the reporting country was the exporter.

### Betweenness Centrality (BC)

BC is a measure that rates the importance of the position of a node or an edge in the network with respect to transport through the whole network. While this is usually done with shortest paths on a graph, here we used a weighted betweenness definition that takes into account the fluxes through edges [22]. For betweenness calculations, the weight of a link is defined as its *resistance* to transport, and one searches for lowest total weight (resistance) paths from a node *m* to a node *n* within the network. The weight of the directed link (*i,j*) is defined as *w_ij_*  = −ln (*Φ_ij_/Φ*_max_), where 
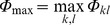
 is the largest flux in the network. Using this logarithmic form, the weights are all positive and additive along a path. If σ*_mn_*(*i*) denotes the number of lowest total weight (LTW) paths from node *m* to node *n* that are passing through *i*, and σ*_mn_* denotes the total number of LTW paths running from *m* to node *n*, the betweenness centrality of node *i* is defined as 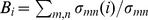 (a similar definition holds for an edge).

### Graph Density

The density of a directed graph *ρ* is given by the ratio *ρ* = *M/*[*N*(*N-*1)], between the number of edges it has, *M*, and the number of edges it could possibly have, *N*(*N*-1), where *N* is the number of nodes. For 2007, we have *N* = 202 and *M* = 13534 giving a graph density of *ρ* = 0.33 (33%), meaning that the graph is not sparse, but rather interconnected.

### Food Flux Model Parameters

Using the fluxes and population sizes directly from the 2007 data, we calculated the fraction *α_i_Π_i/_*(1*+α_i_*)*I_i_* for each country. The ratios obtained were exponentially distributed, with values between 0 and 0.003 [person/US$]. For the backbone countries, they were typically small, less than 0.0002, due to the large fluxes assigned to these countries. The obtained fractions were used to calculate the values of *r_i_* = 1– [*cα_i_Π_i/_*(1+*α_i_*) *I_i_* ], using a constant for the parameter *c* (per person food consumption in a country in a year, expressed in US$). In reality, it varies from country to country but here, as a first approach, we chose a single value representative for the backbone of the IFTN, which indeed, involves countries at similar levels of economic development. This did not affect the results significantly because the fluxes between countries that are not part of the backbone represent a negligible portion of all fluxes in the network. The ranking of countries based on their *R_i_* and *V_i_* values proved to be robust for a wide range of values for the parameter *c* (10< *c* <10^5^ US$). For the simulation results shown we used *c* = 10^4 ^US$. On the other hand, the ranking of the countries proved to be highly sensitive to the distribution of the *α_i_Π_i/_*(1*+α_i_*)*I_i_* fractions; for which, however, we used data from UN databases.

The second model parameter, *β,* was used to define initial conditions for the simulation. It represents the fraction of the exported food that is contaminated. The value of this parameter would depend on the actual contamination; however we use it as a simple multiplying factor which had no effect on the overall ranking. Here we chose *β* = 0.001. Had we chosen for example *β* = 0.01, the values in Table 1 would have been 10 times larger, but there would have been no other changes.
