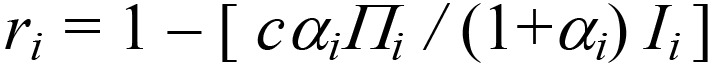# Correction: Complexity of the International Agro-Food Trade Network and Its Impact on Food Safety

**DOI:** 10.1371/annotation/5fe23e20-573f-48d7-b284-4fa0106b8c42

**Published:** 2012-10-30

**Authors:** Mária Ercsey-Ravasz, Zoltán Toroczkai, Zoltán Lakner, József Baranyi

There was an error in the first equation in the third paragraph of the Spread and Tracing on the IFTN section of Results. The correct equation can be viewed here: